# Heart Development, Coronary Vascularization and Ventricular Maturation in a Giant Danio (*Devario malabaricus*)

**DOI:** 10.3390/jdb6030019

**Published:** 2018-07-21

**Authors:** Olubusola Shifatu, Sarah Glasshagel-Chilson, Hannah M. Nelson, Purva Patel, Wendy Tomamichel, Clay Higginbotham, Paula K. Evans, Gregory S. Lafontant, Alan R. Burns, Pascal J. Lafontant

**Affiliations:** 1 Department of Biology, DePauw University, Greencastle, IN 46135, USA; ozs2yy@virginia.edu (O.S.); sarahachilson@gmail.com (S.G.-C.); hannahnelson_2018@depauw.edu (H.M.N.); purvapatel_2019@depauw.edu (P.P.); wendytomamichel@depauw.edu (W.T.); clayhigginbotham@depauw.edu (C.H.); pevans@depauw.edu (P.K.E.); 2 GXP Matrix, Bloomfield, NJ 07003, USA; greg_lafont@yahoo.com; 3 College of Optometry, University of Houston, Houston, TX 77204, USA; arburns2@central.uh.edu

**Keywords:** giant danio, *Devario*, zebrafish, *Danio*, heart, coronary, vessels, development, breeding

## Abstract

Giant danios (genus *Devario*), like zebrafish, are teleosts belonging to the danioninae subfamily of cyprinids. Adult giant danios are used in a variety of investigations aimed at understanding cellular and physiological processes, including heart regeneration. Despite their importance, little is known about development and growth in giant danios, or their cardiac and coronary vessels development. To address this scarcity of knowledge, we performed a systematic study of a giant danio (*Devario malabaricus*), focusing on its cardiac development, from the segmentation period to ten months post-fertilization. Using light and scanning electron microscopy, we documented that its cardiovascular development and maturation proceed along well defined dynamic and conserved morphogenic patterns. The overall size and cardiovascular expansion of this species was significantly impacted by environmental parameters such as rearing densities. The coronary vasculature began to emerge in the late larval stage. More importantly, we documented two possible loci of initiation of the coronary vasculature in this species, and compared the emergence of the coronaries to that of zebrafish and gourami. This is the first comprehensive study of the cardiac growth in a *Devario* species, and our findings serve as an important reference for further investigations of cardiac biology using this species.

## 1. Introduction

Cardiovascular diseases remain the number one cause of death in the Western world. They encompass adult conditions such as atherosclerosis, hypertension, myocardial infarction, and cardiomyopathies of various etiologies [1]. They also include a number of cardiac congenital defects that cause significant pathologies in utero, in neonates and infants, resulting in significant morbidities and increase in health care cost. Studies of mammalian and non-mammalian model organisms such as the mouse [2], amphibians [3], and fish [4], have greatly advanced our understanding of the genes and molecular pathways that regulate cardiovascular development, and the mutations that cause cardiac diseases in human. From the appearance of the first cardiac progenitor cells to the formation of a tubular heart, from cardiac looping to valvulogenesis, from chamber morphogenesis to trabeculation, trabecular compaction or non-compaction, early cardiac development in vertebrates proceeds for the most part along many discrete, essential and well documented morphogenic events [5]. Among these processes, time-dependent coronary vascular development is essential to embryonic viability, cardiac growth and maturation in many species. These morphological and functional developments are regulated by modular, and conserved molecular networks.

Among model organisms, many fish species present unique advantages for developmental studies. Established models such as the zebrafish *(Danio rerio)* and medaka *(Oryzias latipes*) are readily amenable to genetic manipulations [6,7,8,9]. Two other important factors in fish models are the external development of the fertilized eggs as well as the natural transparency of the embryos and early larvae which facilitate visualization and imaging over relatively long periods. With the availability of wide ranging and ever increasing molecular tools, these fish species have become important model organisms used in the investigations of the cellular and molecular events underpinning cardiac development, maturation, and regeneration [10,11,12,13,14]. Along with the zebrafish, studies in other fish species have also contributed to the understanding of key developmental processes in the heart [15,16]. The study of fish reveals the functional and structural diversity of their hearts [17,18,19], and has provided insights into the evolutionary mechanisms that underlie this diversity. 

The *Devarios*, including *Devario aequipinnatus* and *Devario malabaricus* (DM), are cyprinid species closely related to the zebrafish [20]. They are members of the danioninae subfamily that display sizes markedly greater than that of *danios* such as the zebrafish [21]. Long considered members of the genus *Danio*, their phylogenies have been revised; these species have since been reclassified as one of now over 40 known *Devario* species [22,23]. Because of their size, both of these *Devarios* have been historically described and referred to as giant danios. Studies including these two species encompass adipose tissue distribution [24], olfactory bulb morphology [25], and phylogenetics [20], and have also elucidated cellular mechanisms of vision [26,27,28,29,30], swimming and biomechanics [31,32,33]. Even though more work is needed documenting the phenotypic differences between these two *Devario* species, a number of studies have directly or indirectly compared various aspects of the biology of the zebrafish and giant danios. The breeding and development up to 65 days post-fertilization of *Devario aequipinnatus* isolated from Meghalaya, India, has been described [34]. The growth rates of zebrafish and *Devario cf aequipinnatus* are significantly different [35] with the size of the giant danio doubling that of the zebrafish by four weeks post-fertilization.

To date there have been few investigations of the cardiovascular biology of *Devario* species. Studies in our lab have shown that the heart of an adult giant danio (*D. cf aequipinnatus*) possesses robust regenerative capacities similar to those seen in the zebrafish [36]. Recently, we reported on the use of lectins as histochemical tools to study cardiac biology in giant danio and zebrafish [37]. However, little is known regarding the cardiac development, growth and maturation of *Devario* species. This current report aims at filling this gap in knowledge and at extending the usefulness of *Devario* species for interrogating cardiac biology. The findings reported in this manuscript describe the heart development of a *Devario* species (*D. malabaricus*), establish the timing of key cardiac morphogenic events, the growth of the species under different environmental conditions, the emergence of its coronary vasculature, and the maturation of its ventricular myocardium.

## 2. Materials and Methods

### 2.1. Identification of the Giant Danio Devario Malabaricus

DNA was isolated from adult giant danios using Zymo’s Quick-DNA™ Universal Kit (Zymo Research, Irvine, CA, USA). The cytochrome c oxidase subunit I (COI) barcode marker was amplified using previously published sets of primers: LCO1490A and HCO2198A [38] and Fish F1 and Fish R1 [39] PCR products were sequenced using a CEQ 8000 Genetic Analysis System (Beckman Coulter, Pasadena, CA, USA) and compared to published sequences for giant danios, with the COI sequences most closely matching the *Devario malabaricus* (DM).

### 2.2. Animals Breeding and Rearing

Adult giant danio DM from Segrest Farms (Gibsonton, FL) were first raised in 10 or 20 gallon tanks at a density of 1 to 2 fish per gallon at room temperature (25 °C). All fish were kept on 14/10 h day/night light cycles. Experimental procedures were approved by the Committee for the Care and Use of Laboratory Animals at DePauw University. After 1 to 3 weeks giant danio DM breeders were transferred into 10 L tanks on an Aquatic Habitat system with 4 to 8 fish in each tank that included females and males. DM were acclimated to 28.5 °C. The pH of the system water was maintained at an average of 7.00, and the water hardness at an average of 800 µS with automated water exchange of 10% per day. Adult DM were fed primarily Zeigler Adult Zebrafish Diet (Pentair, #AH271, Apopka, FL, USA) twice a day. In preparation for breeding, DM feeding regimen was changed to live brine shrimp larvae hatched from artemia cysts (INVE Aquaculture Inc, Salt Lake City, UT, USA) and frozen bloodworms (San Francisco Bay Brand, Newark, CA, USA). Female DM were monitored for noticeable abdominal fullness indicating the presence of eggs and readiness for breeding.

For breeding, 10 gallon tanks were setup with Top Fin Premium Aquarium Gravel (#5132022, Petsmart, Phoenix, AZ, USA) that were laid loosely packed as a single layer at the bottom. Each tank was filled with approximately 15 cm of system water at 28.5 °C. A clear hole-punched plastic divider separated the tank in half. Two to four colorful plastic plant simile were added to the tanks. Typically, two males and three females were placed on opposite sides of the tanks between 4 and 6 p.m. the evening before, with the light in the room turning off at 10 pm. The temperature in the tanks slowly equilibrated with the room temperature of 25 °C during the night. Two sets of light were used to simulate dawn and full daylight. A small 10 watt, 580 lumen TE spiral light bulb (General Electric, Boston, MA, USA) at the corner of the aquatic room turned on at 7:30 am simulating dawn, and the full set of lights in the room turned on at 8:00 am simulating morning. Ten minutes before 8:00 am, the plastic separators between males and females were removed. Tanks were then inspected subsequently every 20 min for the presence of eggs. In some experiments a single male and single female were used per tank. When breeding was successful, pairs or groups of DM were allowed to rest for one to two months before breeding was attempted. 

Fertilized eggs were collected and transferred into pyrex plates at 50 to 100 embryos per plates and kept in 0.5X E2 embryo water containing 0.5 mg/L methylene blue [40] and were kept in an incubator set at 28.5 °C until the larvae had hatched. Early larvae were kept in 1.5 or 3 L tanks at 28.5 °C in a Percival I-36LLVL environmental chamber with Intellus controller (Percival, Perry, IA, USA), until they were transferred to the Aquatic Habitats system. Larvae were fed paramecium beginning at day 5 and up to 2 weeks, and with Hatchfry Encapsulon, 30 μm microparticles larval diet (Argent Aquaculture, Redmond, WA, USA) from the first to the fourth week. Larvae were transitioned to a combination of brine shrimp hatched from artemia cysts (INVE Aquaculture Inc, Salt Lake City, UT, USA), 150–200 μm, and 250–450 μm Larval AP100 diet (Zeigler, Gardners, PA, USA). By 6 to 8 weeks late larvae and early juvenile DM were fed primarily crushed or intact adult Zeigler Zebrafish diet, supplemented with larval brine shrimp.

Adult Tg(fli1a:EGFP)y1 zebrafish fish were obtained from ZIRC raised and bred according to published protocols. Zebrafish embryos obtained from Dr. Michael Chang (University of Pittsburgh) and Dr. Saulius Sumanas (University of Cincinnati) were also used in these studies. Blue gouramis, also known three spot gourami, were obtained from thatfishplace.com (Lancaster, PA, USA), and Segrest Farms (Gibsonton, FL, USA) raised at room temperature (25 °C in 10 gallon tanks).

### 2.3. Morphometric Analysis

At predetermined time intervals fish were euthanized in 0.2% buffered MS Tricaine (Sigma-Aldrich, St. Louis, MO, USA) in deionized water. When opercular movements ceased, body weight (BW) and standard length (SL) were measured as previously described [36] using an ADAM PW124 analytical Balance (Oxford, CT, USA), and a Johnson 1889-0600 digital caliper (Johnson Level, Mequon, WI, USA). Fish were held ventral side up in a slit cut in a wet sponge block. Using a Leica ZOOM 2000 dissecting microscope (Leica Microsystems, Bannockburn, IL, USA), the skin, pectoral muscles, and the pericardium were gently dissected midline. The heart was quickly removed by gently pulling and severing the ventral aorta proximal to the bulbus, lifting the ventricle and severing the atrium while preserving the atrioventricular junction. The heart was briefly rinsed in 1X PBS to clear out the luminal blood. The heart was drained of excess buffer, weighed, and transferred to ice cold-fixative (500 μL of PBS buffer containing 4% paraformaldehyde) for 3 h or overnight at 4 °C. Small larvae were imaged and measured using a Leica S6D dissecting microscope equipped with a Leica MC170 HD camera (Leica Microsystems, Bannockburn, IL, USA), or a Nikon SMZ 1000 dissecting microscope (Nikon Instruments Inc., Melville, NY, USA) equipped with a Spot Insight QE camera (Diagnostic Instruments Inc., Sterling heights, MI, USA).

### 2.4. Wholemount Lectin Staining and Immunostaining Procedures

For whole-mount studies, following fixation in 4% paraformaldehyde, the hearts or larvae were washed in 1X PBS and permeabilized with 0.5% Triton X-100 in 1X PBS overnight at 4 °C. The hearts were washed and stained immediately. Fluorescein-conjugated and rhodamine-conjugated BS lectin, Con A, or wheat germ agglutinin (WGA) (Vector Laboratories, Burlingame, CA, USA) were used as previously described [37]. Hearts and larvae were incubated wholemount in Cellstar 24 well cell culture plates (Santa Cruz, Biotech, Santa Cruz, CA, USA) overnight at 4 °C using in 400 μL of lectin-containing Tris buffer saline (50-mM Tris, 150-mM NaCl, 1-mM CaCl2, 1-mM MgCl_2_, pH 7.6) at the following final lectin concentrations: BS lectin (1:400, 5 μg/mL), Con A (1:1000, 2 μg/mL), WGA (1:1000, 2 μg/mL). Following lectin incubation, the hearts were washed in PBS, and cell nuclei were stained with Hoechst stain (Invitrogen, Eugene, OR, USA), wash in 1X PBS. For embryonic myosin staining, embryo and early larvae were fixed and permeabilized as described above. The samples were then washed in PBS and blocked with 3% BSA in PBS for 2 to 4 h. Embryos and early larvae were incubated in MYH1/F59 mouse monoclonal antibody at a final concentration of 2 μg/mL (sc-32732, Santa Cruz Biotech, Santa Cruz, CA, USA) in blocking buffer for 2 days at 4 °C. Hearts were washed in 1X PBS three times and incubated in FITC-conjugated goat anti-mouse secondary antibody at 1:1000 dilution (F0257, Sigma-Aldrich, Saint Louis, MO, USA) for 24 h, and washed 3 times in 1X PBS, prior to imaging.

For section staining, fixed hearts were washed in PBS and transferred to PBS containing 30% sucrose for cryoprotection overnight at 4 °C. The next day, the hearts were washed in 1X PBS and embedded in 13-mm-diameter aluminum seal cups (Wheaton, Millville, NJ, USA) filled with freezing medium Tissue-Tek O.C.T. (Ted Pella, Torrance, CA, USA). The samples were frozen, and stored at −80 °C. The hearts were sectioned 10 μm thick and mounted on TruBond 360 (Electron Microscopy Sciences, Hatfield, PA, USA) or Superfrost Plus slides (Fisher Scientific, Waltham, MA, USA).

### 2.5. Tissue Imaging and Image Analysis

For imaging, adult and larval hearts, or embryos were mounted in glass-bottom tissue culture dishes (MatTek, Ashland, MA, USA) in 2% low-melting agarose (Sigma-Aldrich, Saint Louis, MO, USA). The hearts were oriented in a plane that projected a lateral view. Ventricular length consisted of a line connecting the apex to the base of the ventricle, at the midpoint of the base of the bulbus. Stained tissues were imaged using a Zeiss AxioImager M equipped with an ApoTome.2 for optical sectioning (Zeiss, Göttingen, Germany) or a Nikon A1R confocal laser scanning microscope (Nikon Instruments, Melville, NY, USA). Calculation of vascular density was performed as previously described [37] from maximum intensity projections obtained using Fiji [41]. Maximum intensity projections of image stacks were displayed on a Dell 17-inch monitor and overlaid with a point-counting grid with 280 equidistant point intercepts. Two to three fields per heart were evaluated. Vascular density was calculated based on the fraction of point intercepts landing randomly on vessels to the total number of point intercepts covering the projected field.

### 2.6. Scanning Electron Microscopy

For imaging by scanning electron microscopy DM hearts were fixed in 2.5% fresh glutaraldehyde in 100 mM cacodylate buffer overnight at 4 °C. DM hearts were then washed in cacodylate buffer sectioned sagittally using a microtome blade and dehydrated in alcohol gradients. Alcohol was subsequently substituted with increasing concentrations of hexamethyldisilizane (HMDS, Sigma-Aldrich, Saint Louis, MO, USA) in a fume hood. Following three exchanges in 100% HMDS, the hearts were dried overnight before they were coated with 80% palladium-20% gold using a Polaron E5100 sputter coater (Quorum Technologies Ltd., East Sussex, UK) and imaged on a JEOL 5800LV at 15 kV (JEOL USA, Peabody, MA, USA).

### 2.7. Statistics

Data are expressed as means and standard errors of means (SEM). Welch *t*-test was used to compare high density versus low density reared fish at different time points. A one-way ANOVA followed by a Tukey post hoc test was used to analyze growth of larvae during the first four week periods. A *p* value of < 0.05 was considered significant. Linear regression and parametric curve fitting was performed using R (R Development Team 2018, R Foundation for Statistical Computing, Vienna, Austria) and GraphPad Prism 7 (GraphPad, La Jolla, CA, USA).

## 3. Results

### 3.1. Cardiac Development in the Giant Danio Devario Malabaricus

We used COI barcode sequences to identify, breed, and establish a colony of the giant danio *Devario malabaricus* (DM), a closely related species to the *Devario aequipinnatus* and *Danio rerio* (Supplemental Figure S1). We studied the development and growth of this species from larval, through juvenile and adult stages in over 600 fish, and have documented cardiac development and coronary vascularization.

### 3.2. Embryonic Stage (Fertilization to 1.5 Days Post-Fertilization)

**Figure 1:**
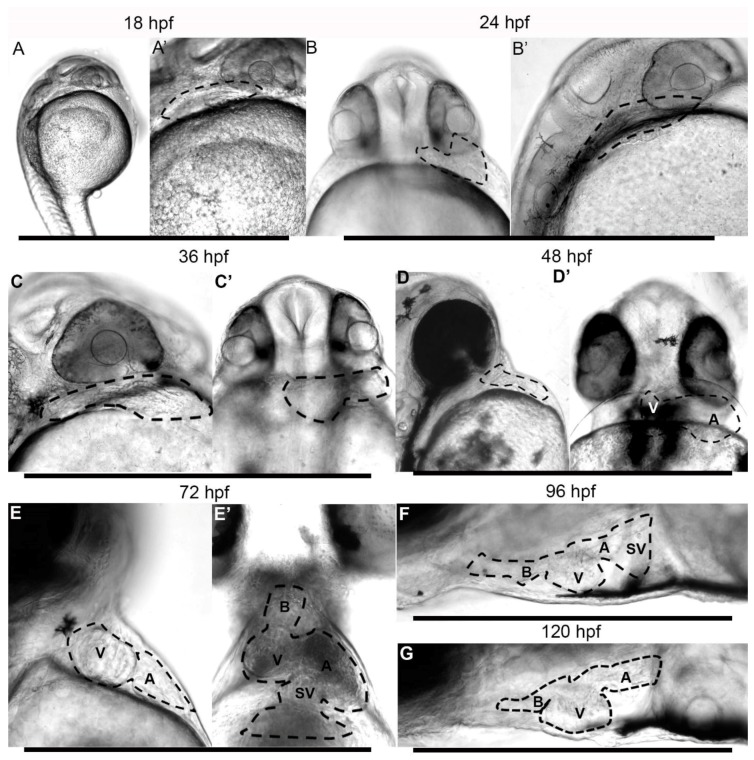
Morphogenesis of the giant danio *Devario malabaricus* (DM) developing Heart. Representative difference interference contrast (DIC) image of a sagittal view of a newly hatched DM larvae (**A**). The heart is tubular, laying posterior to the developing eye, extending dorsally, displaying rhythmic and coordinated contraction (Supplemental material movie 1). The tubular heart (outline) at 18 hpf at higher magnification (**A’**). Representative DIC image of a ventral view of a 24 hpf DM larvae (**B**) with the tubular heart (outline) projecting to the left. Sagittal view of the same heart laying posterior to eye, extending dorsally (**B’**). Representative DIC image of a sagittal view of a 36 hpf larvae showing a primarily tubular heart (**C**,**C’**). Representative DIC image of a sagittal view of a 48 hpf larvae showing a segmented heart (**D**). From a ventral view, the ventricle can be seen to have shifted to the right and the atrium remaining on the left (**D’**), the atrioventricular junction approaching the midline of the anteroposterior (AP) axis. Representative DIC image of a sagittal view of a 72 hpf larvae showing segmented heart (**E**) with the ventricle occupying a more rostral position. From a ventral view, the ventricle can be seen to have migrated to the right and the atrium remaining on the left ((**E’**), and Supplemental Material movie 3), with the atrioventricular junction occupying the midline of the AP axis. At 96 hpf (**F**) and 120 hpf days (**G**), the heart has assumed its final position, with the atrium is located posterior dorsal to the ventricle, and the bulbus anteriorly (Supplemental Material movie 4). A = atrium, B = bulbus, SV = sinus venosus, V = ventricle.

First, examination by DIC microscopy of embryos raised at 28.5 °C revealed the presence of a beating heart as early as 18 h post-fertilization (hpf) (Figure 1A,A’, and Supplemental Material movie 1). At that early stage and at 24 hpf (on left sagittal projection) the beating heart was located posterior to the developing eye and inferior and subjacent to the otic vesicle (Figure 1B). The ventral projection shows the heart at the far anatomical left of the midline and at the anterior margin of the yolk sac (Figure 1B’). The longitudinal axis of the heart was nearly perpendicular (90°) to the anterior-posterior midline of the embryo. While cardiac contractions were present at 18 hpf, we did not observe the presence of circulation, or evidence of circulating cells within the beating heart, the Ducts of Cuvier, on the surface of the egg yolk or the fish trunk. The first visual evidence of circulation appeared shortly before or at 21 hpf (Supplemental Material movie 2). At that time, circulating blood cells could be seen transiting through the heart, the Ducts of Cuvier and in the major trunk vessels toward the fish tail superiorly and returning from the tail inferiorly. In the next 12 to 24 h, the heart remained on the left of the giant danio, displaying a tubular form wider at the margin, and narrower toward the midline. The angle from the antero-posterior midline of the embryo and early larva decreased progressively as the heart tube began segmentation and migrated toward the midline between 24 and 48 hpf (Figure 1C,C’).

### 3.3. Early Larvae (36–72 h Post Fertilization)

Hatching marks the transition from the embryonic and the larval stage, yet the time of hatching is variable and depends in part on environmental factors. Under our rearing conditions, all viable embryos hatched by 36 h. Hatching and straightening of the early embryo caused marked relocation of the heart. By 48 hpf, the anatomical segmentation of the heart was established, and the ventricle had begun to migrate to the right, crossing the midline (Figure 1D,D’). A thickened bulbus could not be observed at that time. By 72 hpf the ventricle shifted further to the right of the midline; the atrium remained mostly on the left of the midline, but had begun to shift caudally (Figure 1E,E’, and Supplemental Material movie 3). By 96 hpf, the atrium had rotated dorsally, as the ventricle occupied the ventral aspect of the thoracic cavity. The heart in its final orientation was observed by five days post-fertilization (Figure 1F,G, and Supplemental Material movie 4).

**Figure 2:**
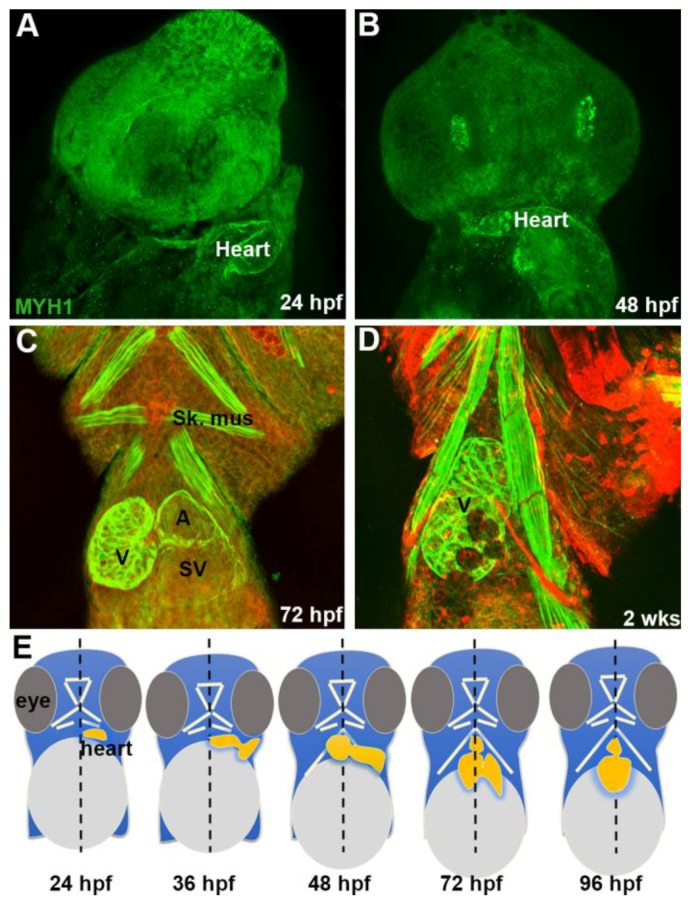
Myosin expression and giant danio DM heart morphogenesis. Myosin (MYH1) immunoreactivity of DM heart at 24 hpf (**A**), the fish rotated 45 degrees from the AP midline. MYH1 immunoreactivity in a 48 hpf DM larvae (**B**) showing segmentation of the heart with the ventricle and atrium shifted more toward the midline compared to 24 hpf. MYH1 immunoreactivity in a 72 hpf DM larvae showing pectoral and jaw skeletal muscle, and the segmented heart (ventricle, atrium, and sinus venosus) with the ventricle shifted to the right of the AP midline plane and the atrium remaining on the left (**C**). Note abundant luminal cardiac trabeculae projecting into the center of the ventricle while they are absent in the atrium and sinus venosus. Myosin staining of DM heart at 2 wpf (**D**), framed by pectoral and jaw muscles. Only the ventricle can be observed from the ventral view as the atrium is rotated posteriorly. (**E**) Graphical representation of morphogenesis and anatomical changes to the developing heart in the first 4 dpf based on DIC live imaging and myosin staining. A = atrium, B = bulbus, SV = sinus venosus, sk. mus = skeletal muscle, V = ventricle.

The initiation of contraction of the heart by 18 hpf suggested the development of the contractile apparatus and the presence of myosin. Myosin immunostaining was detected in the tubular heart at 24 hpf (Figure 2A), within the segmented heart, as distinct atrial and ventricular chambers formed at later stages (Figure 2B), in the ventricle as it assumed a rostral position, right of the anterior-posterior midline, and in the atrium as it came to lie in a more caudal position left of the midline (Figure 2C). By 7 days, the entire ventral projection of a trabeculated ventricle in situ was observable, and only a small aspect of the atrium was visible outside the ventricular margin. A final rotation had occurred that located the atrium on the dorsum of the ventricle (Figure 2D). An illustrated summary of the morphogenesis of the DM heart from DIC and immunostaining during the first four days is provided (Figure 2E).

### 3.4. Growth Parameters in Post Embryonic DM

**Figure 3:**
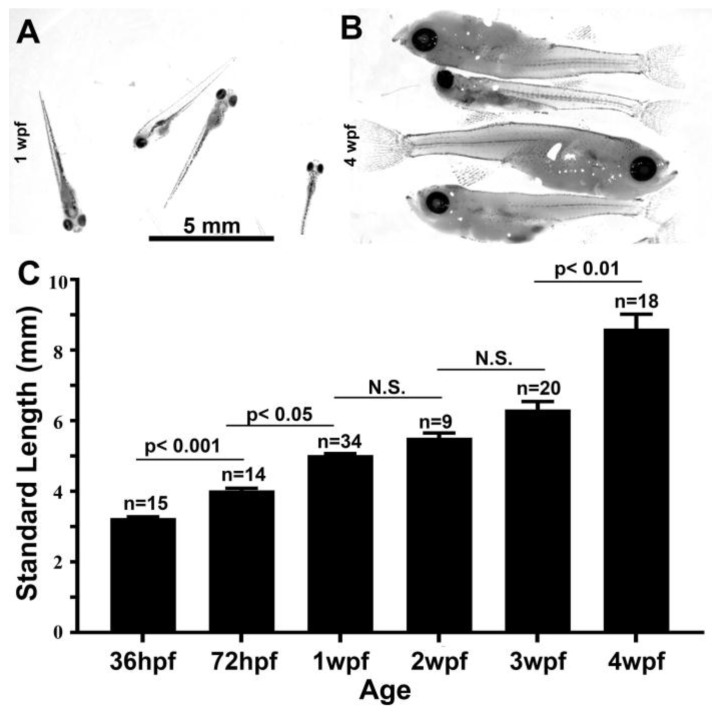
Growth of the early DM larvae. Representative images of 1 week post-fertilization (wpf) giant danio (**A**) and 4 wpf early larvae (**B**). Change in standard length of giant danio larvae from 36 hpf to 4 wpf (**C**). Data represent means and standard error from four independent experiments, analyzed using ANOVA, with *p* < 0.05 considered significant. Scale bar = 5 mm.

**Figure 4:**
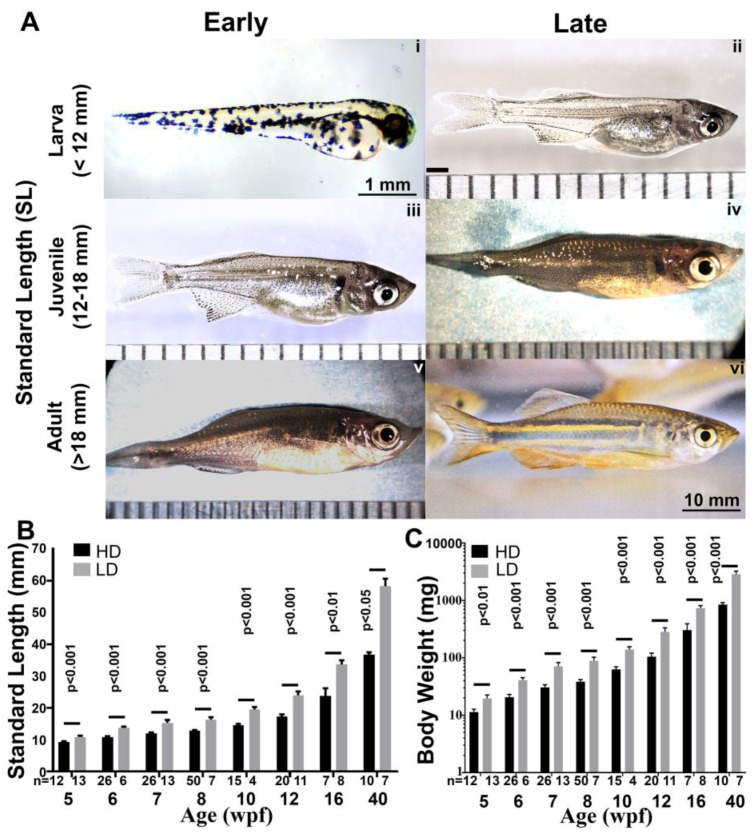
Rearing-density effects on the growth and maturation of the giant danio DM. Images of representative DM early larvae (**A-i**) and late larvae (**A-ii**) showing dramatic growth and changes in phenotype. In addition to the abundant melanophores seen in the early larvae, iridophores are prominent in the late larvae as well as the early juvenile (**A-iii**) giving the fish a distinctly silvery hue. Progressive phenotypic changes, increase in size and the appearance of longitudinal bands of xanthophores characterizes the late juvenile DM (**A-iv**) and early adult (**A-v**) and late adult (**A-vi**). Standard length (SL) measured in low and high density reared fish from 5 to 40 weeks post-fertilization (wpf), and showing differential but linear growth in length (**B**). Body weight (BW) measured in low and high density reared fish from 5–40 wpf showing differential but logarithmic increase in BW (**C**). Scale bars are 1 or 10 mm, and ruler units are 1 mm intervals. SL and BW of high and low density raised fish are analyzed using Welch two sample *t*-test where *p* < 0.05 is considered significant.

We used staging parameters developed for the zebrafish as a framework to study the growth of the giant danio DM. First, we studied larval growth (Figure 3A,B) from hatching at 36 hpf to 4 weeks post-fertilization (wpf). During that time period, the larvae experienced rapid growth starting at and SL of 3.2 mm at hatching and reaching 8.6 mm by 4 weeks, doubling by 3 wpf (Figure 3C). Next, we investigated the overall growth of the giant danio from mid-larval stage to adulthood (Figure 4A), and determined the effects of rearing density on growth parameters. For these experiments, larvae were separated at 3 wpf into two groups: a low density reared (LDR, 1–2 fish per liter) and a high density reared (HDR, 5–10 fish per liter), with the recording of growth parameter measurements starting at 5 wpf. Small but significant differences in SL could be detected in fish reared under these conditions, with the LDR fish SL measuring an average of 11 mm while HDR fish measured 9 mm, approximately an 18% difference between the two groups (Figure 4B). From 6 weeks to 10 months (40 wpf) old, the difference in fish SL was sustained, with LDR fish SL values ranging from 30 to 60% higher than HDR fish. Growth during this period culminated in an average SL of 58.3 mm in the LDR and a SL of 36.7 mm in HDR at 10 months. As a result of the differential growth, LDR DM reached the juvenile stage (SL >12 mm) between 5 and 6 weeks, while fish grown at higher densities reached the same stage between 7 and 8 wpf. LDR DM reached adult size (SL >18 mm) between week 8 and 10, compared to HDR fish that reached adult size between 12 and 16 wpf. In adults the difference in SL for the LDR and HDR fish was approximately 10 mm at 16 wpf; the difference in SL had doubled at 40 wpf to approximately 20 mm.

Differential growth pattern was also observed in the body weight of fish in the two groups. At 5 weeks BW in the LDR averaged approximately 20 mg, compared to 11 mg in the HDR-DM. From 5 to 16 wpf, the BW in both groups increased at a high rate. The BW increased more than 35 times in the LDR fish compared to 30 times in the HDR fish. Between 16 and 40 wpf, the BW in HDR-DM approximately tripled, while the LDR-DM nearly quadrupled (Figure 4C).

### 3.5. Ventricular Growth in Post Embryonic DM

**Figure 5:**
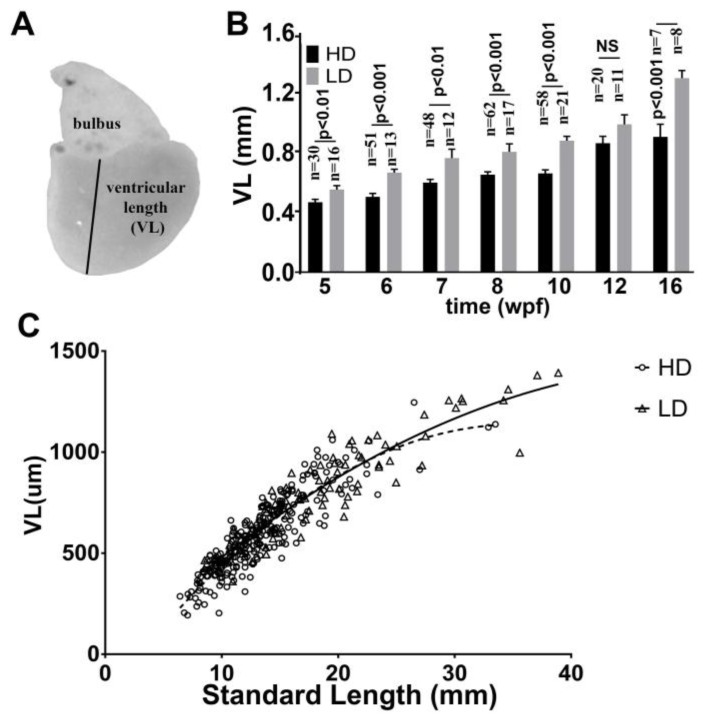
Rearing-density effects on the ventricular growth of giant danio DM. Image of an excised adult DM heart (**A**). The vertical line drawn from the middle of the bulbus at the base of the ventricle to the apex defines the ventricular length (VL). VL of DM measured at high and low density rearing conditions from 5 to 16 wpf (**B**). VL of high and low density raised fish are analyzed using Welch two sample *t*-test where *p* < 0.05 is considered significant. Scattered plot with polynomial fits of DM VL plotted against their standard lengths (SL) showing a positive correlation (**C**).

Next, we determined the effects of rearing conditions on ventricular length (VL) as an index of cardiac growth (Figure 5A), and the relationship of VL to SL. We measured VL of fish from 5 wpf when the ventricle could be easily and consistently removed, until 16 wpf. We found that overall VL in this period increased in both fish cohorts from approximately 0.5 mm at 5 weeks to greater than 1 mm at 16 weeks. Moreover, significant differences in ventricular growth were observed between LDR and HDR fish over the period studied, similar to that recorded for SL. In the LDR fish VL increased from 0.53 mm at 5 weeks to 1.2 mm at 16 weeks. In the HDR fish VL rose from 0.45 to 0.87 mm during the same period (Figure 5B). A close correlation was found between VL and SL in both the HDR and LDR ventricles (Figure 5C).

### 3.6. Coronary Vascularization and Ventricular Maturation in the Post-Embryonic DM

**Figure 6:**
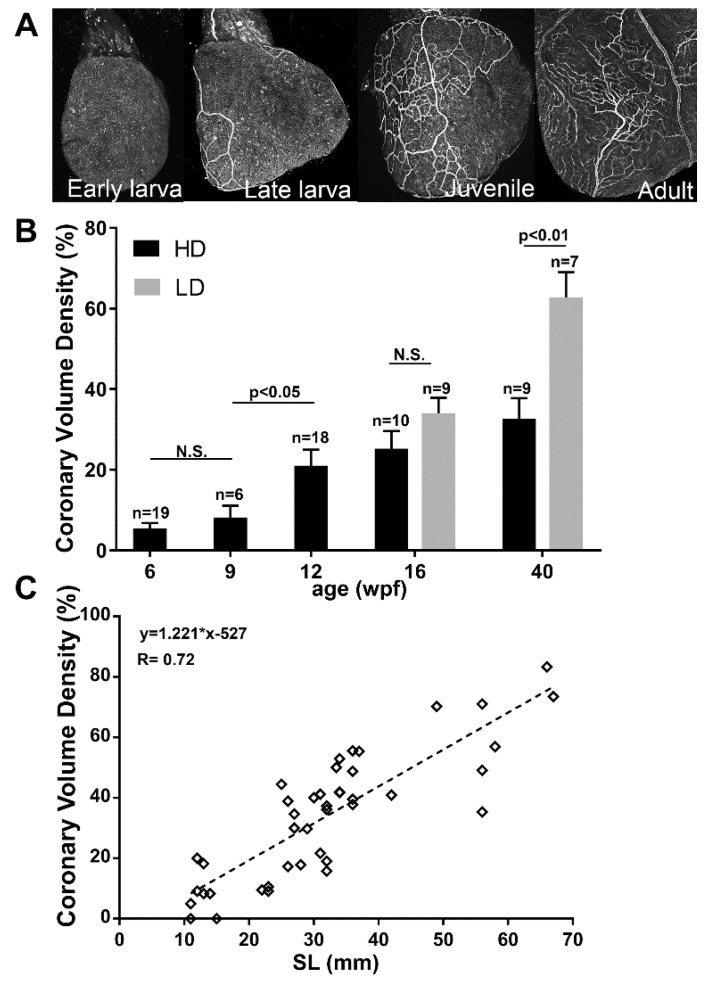
Coronary vascular development in the giant danio DM Heart. Ventral view of a representative B.S. lectin stained heart of an avascular early DM larvae ((**A**), panel 1), a late larvae with a developing coronary vascular network ((**A**), panel 2), further expansion of the network in a juvenile ((**A**), panel 3), and in an adult DM ((**A**), panel 4). Quantitation of coronary vascularization of the heart from 6–40 wpf, and the effects of rearing density (**B**). Coronary volume density (CVD) of high and low density raised fish are analyzed using Welch two sample *t*-test where *p* < 0.05 is considered significant. Scattered plot of CVD of giant danio DM over standard length (**C**) showing a positive correlation.

Coronary vascularization is crucial or essential for oxygenation, growth and maturation of most vertebrate hearts. From hatching and through most of the larval stage, the giant danio DM heart remained avascular. The first coronary vessels appeared in the late larval stage and coincided with a subsequent transition into the juvenile stage. Once initiated, a steady expansion of the coronary vascular network was observed (Figure 6A). Rearing density had a significant effect on coronary vessel expansion, with coronary vascular density markedly higher in LD-reared compared to HD-reared DM by 16 wpf. The growth of coronary vessels culminated in coronary vascular density of LD-reared DM that approximately doubled that of HD-reared DM by 40 wpf (Figure 6B). The DM ventricle remained avascular in fish less than 10 mm in SL. Indeed, the first observed vessels arose between 10.5 and 12 mm in SL (Figure 6C). Moreover, the overall coronary vascular density increased linearly with SL, however, there were marked variations between fish of similar size.

**Figure 7:**
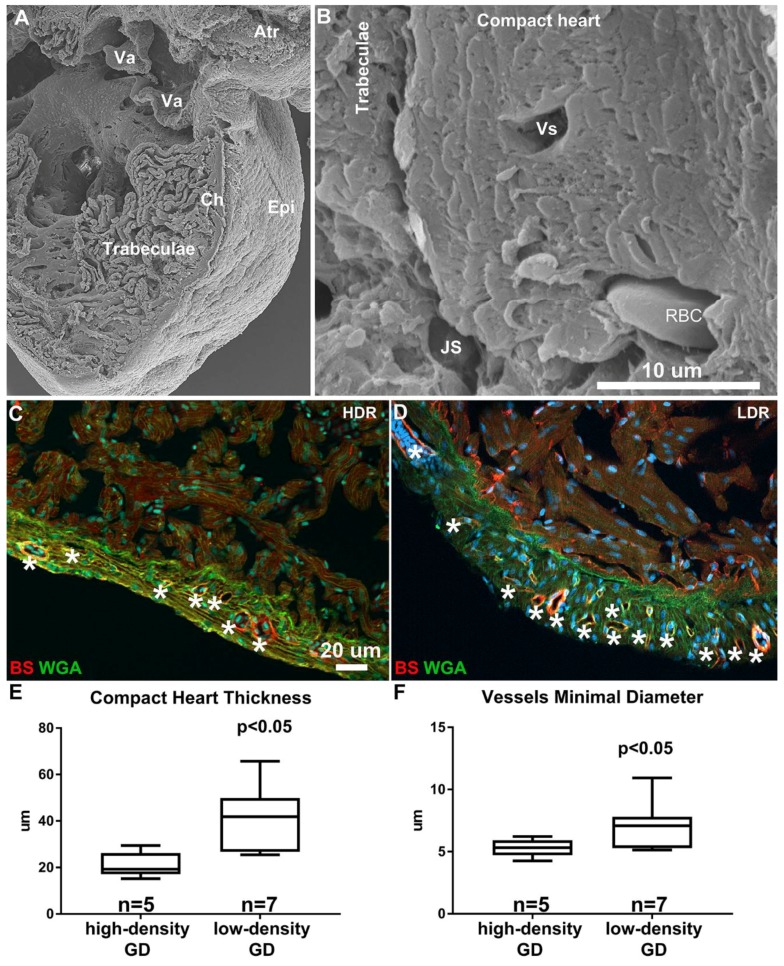
Structure of the Adult giant danio DM. Scanning electron micrograph of adult giant danio heart illustrating the ventricle and the dense and highly trabeculated spongy heart, the thin epicardium (Epi) and relatively thick compact heart (Ch). The atrioventricular valves (Va) and part of the atrium (Atr) can also be seen (**A**). Higher magnification image (**B**) of the compact heart showing multiple layers of cardiac myocytes forming a thick myocardium, traversed by numerous coronary vessels (Vs), one of which contains a red blood cell (RBC). The junctional space (JS) between the compact and trabeculae is noted. Scale bar = 10 μm. Image of adult giant danio heart section double stained with wheat germ agglutinin (WGA, green) highlighting the borders of compact heart myocytes, and Bandeiraea simplicifolia lectin (BS, red) labeling endothelial cells of the coronary vasculature in high density (**C**) and low density-reared fish (**D**). Scale bar = 20 μm. Quantitation of compact heart thickness (**E**) and minimal coronary vessels diameter (**F**) in 10-month old giant danio DM. Data analyzed using *t*-test with *p* < 0.05 considered significant.

Coronary vasculature expansion during the adult stage corresponded with an increase in ventricular size parameters. Imaging performed by scanning electron microscopy showed the adult ventricle was composed of a dense network of trabeculae projecting radially into lumen (Figure 7A). An 8 to 14 cardiac myocyte-thick ventricular wall highly vascularized with coronaries of various sizes constituted a compact heart region that reached 50–60 μm in thickness (Figure 7B). Similar to other morphometric measures (SL, VL, coronary vascular density), rearing density also impacted the thickness of the compact heart. Indeed, the compact heart of the HD-raised DM was less thick than that of the LD-raised DM (Figure 7C,D), with the HD-raised DM compact heart thickness averaging 23 μm, while the LD-raised DM was nearly doubled at 42 μm (Figure 7E). In addition, the average minimal diameter of coronary vessels was 5 (range 3–14) in the HD-raised DM compared to 7 mm (range 3–33) in the LD-raised DM (Figure 7F).

### 3.7. Origin of Coronary Vessels in the Giant Danio, Zebrafish and Gourami

**Figure 8:**
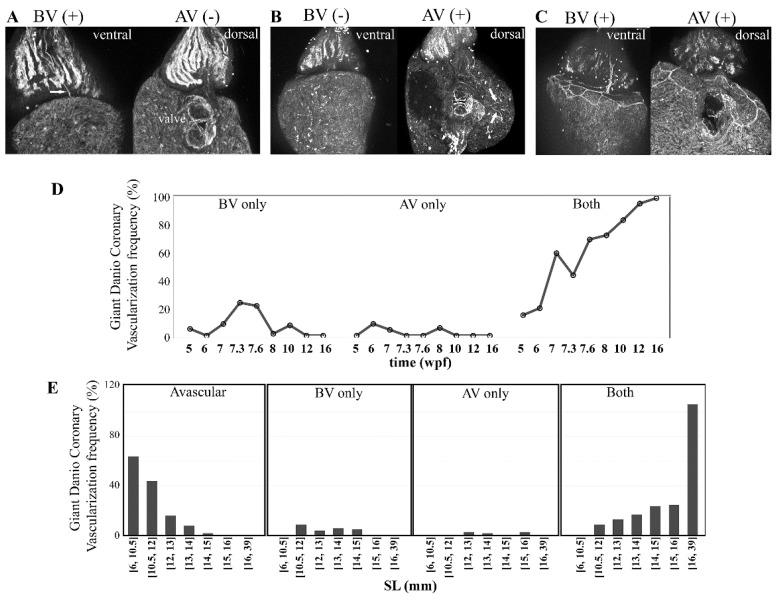
Origin of the coronary vasculature in the giant danio DM. Image of a BS lectin stained heart with a single vessel (arrow) over the ventral surface of the bulbus, in contact with the base of the ventricle, and of the same heart rotated 180 degrees, with no evidence of coronary vessel on the dorsal aspect of the ventricle, or near or around atrioventricular junction ((**A**), left panel). View of a BS lectin stained heart with no identifiable vessel over the bulbus ((**B**), left panel), and the same heart rotated 180 degrees with vessels connected to the atrioventricular junction on the dorsal aspect of the ventricle ((**B**), right panel)**.** Image of a BS lectin stained heart with a single vessel (arrow) over the ventral surface of the bulbus, in contact with the base of the ventricle ((**C**), left panel), and of the same heart rotated 180 degrees, with vessels at the base and dorsal aspect of the ventricle and connected to the atrioventricular junction ((**C**), right panel). Frequency and distribution of coronary vessels over the bulbus only (BV) and the atrioventricular junction only (AV) from 5 to 16 wpf (**D**). Frequency and distribution of coronary vessels over the bulbus (BV) and the atrioventricular junction (AV) over standard length (**E**).

To ascertain the site of initiation of coronary vasculature in the giant danio DM, we screened a subset of 363 late larval and juvenile fish between 5 and 16 wpf, to identify larvae with first vessel over the bulbus and in contact with the base of the heart, or those with first vessel at the AV junction alone. In over a third of larvae (134, 36.9%) the ventricles were avascular, and vessels were observed neither over the bulbus nor at the AV junction. We also found hearts with a first vessel over the ventral aspect of the bulbus and contacting the base of the ventricle (*n* = 26, 7.2%), in the absence of AV junction coronary vessels (Figure 8A). We also observed a number of hearts (*n* = 9, 2.5%) with vessels only present in the AV region (Figure 8B). In the majority of larvae (194, 53.4%), either a single or a pair of bifurcating coronaries were found coursing over the bulbus arteriosus, connected to the base of the ventricle, and were continuous with coronary vessels also present at the AV junction (Figure 8C). In addition, we noted a bias in vessels appearing over the bulbus only, at earlier time points than vessels appearing at the AV junction only (Figure 8D). Temporal differences in coronary emergence were also found within and between the low- and high-density reared DM. 

**Figure 9:**
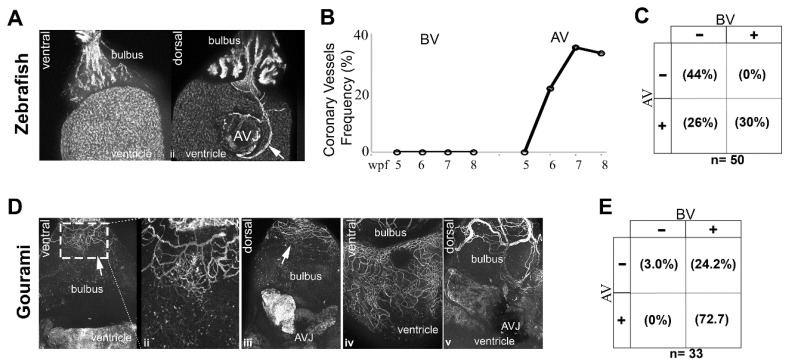
Origin of the coronary vasculature in the blue gourami and zebrafish. Image of a Tg(fli1:EGFP)y1 fish with no identifiable vessel over the ventral aspect of the bulbus (**A-i**), and the same fish rotated 180 degrees with a visible vessel on the dorsal aspect of the ventricle and connected to the inferior aspect of the atrioventricular junction (**A-ii**). Frequency of coronary vessel appearance over the bulbus only (BV), and the atrioventricular junction only (AV) over time in the Tg(fli1:EGFP)y1 fish (**B**). Overall frequency and distribution of coronary vessels in the Tg(fli1:EGFP)y1 between 5 and 8 weeks post fertilization (**C**). Image of a BS lectin stained gourami heart with a vascular plexus over the surface of the cranial aspect of the bulbus (**D-i**) and the vessels at higher magnification (**D-ii**). No vessels are present over the ventricle. The same heart rotated 180 degrees shows the same vessel plexus at the cranial end of the dorsal aspect of bulbus, but no evidence of coronary vessel at or near the atrioventricular junction or the ventricle (**D-iii**) (**B**). Further development of the coronary vasculature of the gourami with coronary vessels investing the ventral and dorsal aspect of the ventricle (**D-iv,v**). Overall frequency and distribution of coronary vessels in the gourami ranging from 40–53 mm in standard length (**E**).

**Figure 10:**
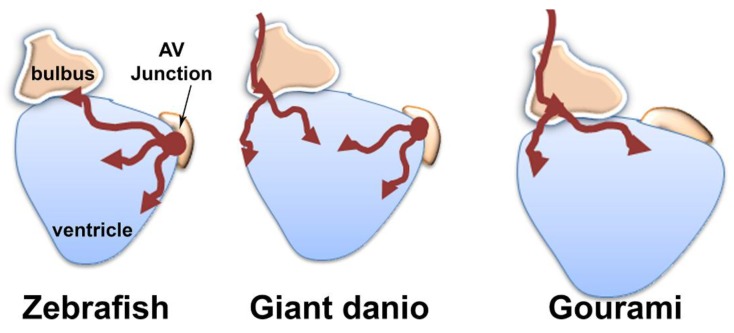
Models of coronary vessel emergence. Coronary vessels emerge first at the atrio-ventricular (AV) junction in the zebrafish, whereas in the giant danio DM, the first coronaries emerge as an extension of a hypobranchial vessel and/or at the AV junction. In the gourami, the first coronaries emerge as an extension of one or many hypobranchials.

Vessels in the zebrafish have been shown to originate first at the AV junction. We screened 50 Tg(fli1a:EGFP)y1 zebrafish between 5 and 8 wpf for comparison with the DM. Our observations were similar to that previously reported, with vessels appearing first at the AV junction (Figure 9A,B). Coronary vessel initiation began at 6 wpf (Figure 9C), or when the zebrafish reached 9 mm in SL. For further comparison, we studied coronary vascularization in blue gourami (*Trichogaster tricopterus*), a species belonging to the *osphronemidae* family, and has shown BS lectin reactivity in coronary endothelium. In the gourami coronary vascularization was not observed before the fish reached 40 mm SL. By contrast to the zebrafish, the first coronary vessels appeared near the cranial end of the bulbus (Figure 9D). Unlike the giant danio where a single vessels course over the bulbus in a cranial caudal direction, and bifurcate toward the base of the ventricle, in the gourami multiple vessels forming an interconnected plexus spread over the proximal bulbus (Figure 9D(i–iii)), progressed and connected to the base of the ventricle. No vessels were identified at the AV junction before the surface of the bulbus and the base of the ventricle were vascularized. (Figure 9D(iii,iv)). A graphical representation of these findings is illustrated in Figure 10.

## 4. Discussion

The data presented here describe the first anatomical and morphological characterization of the developing heart in a *Devario* species, the *Devario malabaricus* (DM). They also represent the first record of overall growth and cardiac maturation of the DM from larva to adulthood under different constraints. To date, nearly 40 *Devario* species have been identified [22,23], however most of the available literature has involved the study of two members of this group: *Devario* cf. *aequipinnatus* [20,21,25,27,28,29,30,35] and *Devario malabaricus* [26,42], both of which have been called giant danios. Among the *Devarios*, these two stripped and barred species have long been identified by unique but markedly overlapping phenotypic characteristics. The studies reported herein pertain to a giant danio and their progenies identified as *Devario malabaricus*.

Comparative morphometric studies between the zebrafish (*Danio rerio*) and a giant danio (*Devario* cf. *aequapinnatus*) have described significantly higher growth rates in the later [21,35]. The overall growth and development of the *Devario aequipinnatus* has been described [34]. The early larval development of *D. malabaricus* from eggs isolated from the wild in Ceylon (Sri Lanka) has also been described [43]. Here we provide the first description of the breeding and overall growth rate of the DM larvae and adult. In the first four weeks post-fertilization, the DM has more than doubled its standard length (3 to 8 mm). This increase parallels that observed in the *D.* cf. *aequipinnatus*, and is higher than that reported for zebrafish [35], suggesting that in addition to reaching much larger sizes, *Devarios* species also have higher growth rates than *danio* species. Similar to the zebrafish, the detection of myosin heavy chain facilitated the temporal study of morphological events in the early development of heart segments. A combination of live imaging and wholemount immunofluorescence allowed us to document the first evidence of a beating tubular heart in a *Devario* as early as 18 hpf, its migration toward the midline, and its chamber morphogenesis by the second day. By the third day clear cardiac segmentations with morphologically distinct ventricular and atrial chamber chambers could be observed, with a final rotation that locates the ventricle rostro-ventrally and the atrium caudo-dorsally. With little variation, these processes are analogous to those observed in zebrafish [6,10,13,44,45]. Whether similar molecular mechanisms orchestrate the DM and zebrafish heart morphogenesis requires further studies.

Several complementary developmental and maturation staging systems for the zebrafish have been proposed [13,46,47]. Developing zebrafish are embryos in the first three to five days and become larvae when they hatch. The transition from larva to juvenile is recognized at 12 mm in standard length, and zebrafish display fully adult phenotypes once they reach 18 mm SL, typically by three months of age. By these measures and under our rearing conditions DM embryos hatch and are larvae by 36 hpf. Subsequently the DM larvae reached the juvenile stage as early as 5 to 6 wpf. The DM adult stage is reached as early as 7 to 8 wpf, a month earlier than the zebrafish. However, while zebrafish phenotypic characteristic at 3 months of age or at 18 mm in SL resembles the adult, the 3 months old or 18 mm standard length DM does not resemble the adult parental pairs. Another index of maturation into adulthood is the ability to reproduce; zebrafish are able to breed at three months post-fertilization. We were unsuccessful at breeding DM at the 18 mm SL or at three months when the fish still appears juvenile. The adult phenotype began to manifest by one year of age or when the DM standard length approached 40 mm. Successful breeding of DM reared in our lab was only possible when the fish were one year old. We cannot rule out that this delay may be the result of suboptimal nutrition or the lack of other essential environmental cues typically available in the wild. Further detailed descriptions of growth characteristics are required for a developmentally appropriate staging system for the giant danio DM.

The acquisition of coronary vasculature and the thickening of the compact heart are among important indices of progressive cardiac maturation. Our studies document the emergence and growth of the coronary vasculature of the DM, and the maturation of the ventricular wall. In mammals, coronary vascularization commences prior to birth, expands in early post-natal age, and is crucial to survival. In fish the time frame of this milestone is relatively protracted (zebrafish) or are absent (medaka). In our studies of the zebrafish, coronary vascularization and the thickening of the compact heart began at 7 wpf and this is consistent with the one- to two-month window previously reported [48]. Similarly, we found that coronary development of the DM was also protracted, but appears as early as five weeks post fertilization in low density reared fish, and later in high density reared fish. This suggests an early temporal need for a vascularized heart in the DM that may be related to the ecological physiology need of a fish inhabiting its native fast streams in the high plateaus of India and Sri Lanka [43]. Interestingly, when assessed by size, the first coronaries in the DM appear at a SL of 12 mm, but appear in the zebrafish at 9.5 mm. These observations suggest that both size and age may regulate the appearance of coronary vessels, and this is consistent with previous observations in the zebrafish. It is possible that these two parameters are independent; however, more controlled studies are required to uncouple these two important factors. In addition to age and size, other factors such as differential levels of activities or social hierarchy in the rearing tanks may need to be considered. Our findings also suggest plasticity in the DM’s abilities to match or constrain its growth to environmental conditions, and demonstrate that rearing density has a major effect on overall growth (as well as coronary vasculature growth) similar to that observed in zebrafish [49].

The origins of coronary vessels in the developing mammalian and non-mammalian hearts are areas of intense investigations. While lineage tracing studies in the mouse show the endocardium and angiogenic sprouts from the sinus venosus [50], as well as vasculogenic blood islands contribute to the endothelial lining of the coronary vasculature [50,51,52,53], the relative contribution of each source is still a matter of debate [54]. Various origins of coronary vessels in fish have been reported. In the dogfish heart, the coronary vascular network appears to proceed from a sinus venosus source via endocardial invagination [55]. More recently a study of the zebrafish showed that the first vessels emerge from a subpopulation of endocardial cells at the atrioventricular junction [48]. Our study identify two possible sources for coronary vessels emergence in the DM. The primary manifestation of coronaries appears as an extension of one pre-existing hypobranchial artery that bifurcates into two vessels (distally over the ventral aspect of the bulbus arteriosus) that connect with the base of the ventricular myocardium. The specific extra-cardiac hypobranchial vessel has not been identified outside the pericardial sac. The secondary manifestation of the first coronary appears as a vessel emerging at the atrioventricular junction, similar to that seen in zebrafish. In this setting, the first coronary vessel may arise from a subset of endocardial cells at the AV. Interestingly, our comparative study of the gourami, a species from a distant clade, we found that their coronary vessels emerge as a plexus of vessels over the proximal bulbus and spreads caudally to make contact with and invest the base of ventricle, in the absence of identifiable AV junction vessels. Taken together, these observations support the notion that fish have developed multiple processes to construct the coronary vasculature required for ventricular growth and maturation. 

In summary, our studies are the first to describe heart development and maturation in a *Devario* species. First, the developing heart of the DM follows morphological changes similar to those described in the zebrafish, another cyprinid, suggesting conservation of these morphometric events. However, the molecular mechanisms orchestrating these morphological changes remain to be determined. Second, cardiovascular development and growth of the coronary vasculature of the DM is responsive to physical and environmental factors. Third, the DM demonstrates a faster growth rate than that reported in the zebrafish and in adulthood it achieves a compact heart thickness two- to three-times greater than that seen in the adult *danio rerio*. Fourth, our findings in the DM and gourami strongly suggest multiple origins of coronary vasculature in teleost fish. Indeed, we have identified two spatially distinct sources of coronary vessels in the giant danio. A major limitation of the current study involves the source of cells from which the coronary vasculature emerges and the nature of these vessels. Molecular markers have been used to identify both coronaries and lymphatics in the zebrafish heart. Our findings are based on the use of lectin histochemistry which depends on endothelial expression of glycoconjugates. Although a robust marker of coronary endothelium in the giant danio, B.S. lectin reactivity does not provide insight into the nature of the emergent coronary vessels. On the basis of their originating loci, it is possible that BV and AV vessels may represent coronary arteries and veins respectively. However further histological studies are necessary to test this hypothesis. Finally, the ability to genetically label and trace these two cell populations is required to ascertain the contributions of these two sources to the developing and mature coronary vasculature of the DM. Despite these limitations, our study markedly extends the usefulness of *Devario* species in the study of cardiac development and maturation. 

## Supplementary Materials

The following are available online at http://www.mdpi.com/2221-3759/6/3/19/s1, Supplemental Figure S1: DM phylogram, Movie S1: DM heart at 18 hpf, Movie S2: DM heart at 21 hpf, Movie S3: DM heart at 72 hpf, Movie S4: DM heart at 96 hpf.
