# Supplementary material for: Heart Development, Coronary Vascularization and Ventricular Maturation in a Giant Danio (Devario malabaricus)
Source: J Dev Biol. 2018 Jul 21;6(3):19. doi: 10.3390/jdb6030019 (PMC6162710; doi:10.3390/jdb6030019)
Supplement: Supplementary file 1 [file jdb-06-00019-s001.zip › JDB GD supplemental data/Supplemental figure S1.pdf]

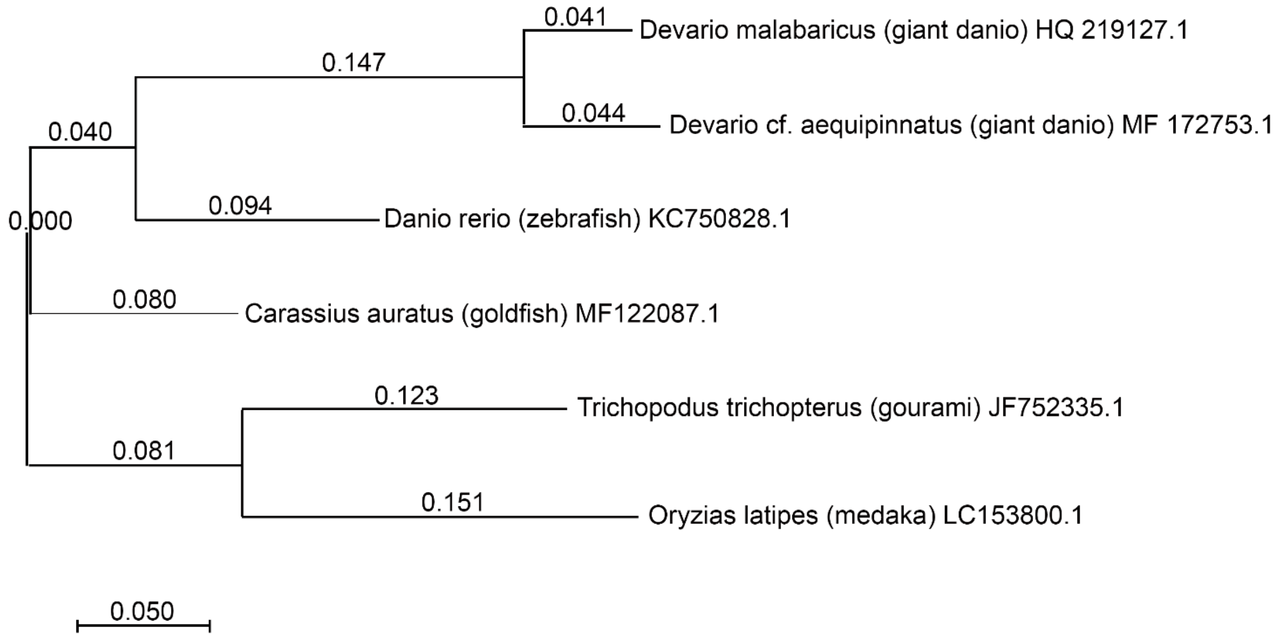

Supplemental Figure 1: Phylogram of giant danios*.* Phylogram of relationships of giant danios (*Devario malabaricus and Devario cf. aequipinnatus), and* select species used in cardiovascular research, inferred by Maximum Likelihood method using the mitochondrial *COI* gene. The tree is drawn to scale, with branch lengths measured in the number of substitution per site (next to the branch). Evolutionary analysis were conducted in MEGA 7. GenBank Accession numbers follow the species; common names are in parentheses.
